# Prevalence and outcome of teenage hospital births at the buea health district, South West Region, Cameroon

**DOI:** 10.1186/s12978-015-0109-5

**Published:** 2015-12-23

**Authors:** Thomas Obinchemti Egbe, Amadeus Omeichu, Gregory Edie Halle-Ekane, Charlotte Nguefack Tchente, Eta-Nkongho Egbe, Jean-Francois Oury

**Affiliations:** Department of Obstetrics and Gynecology, Douala General Hospital, Box 4856, Douala, Cameroon; Faculty of Health Science, University of Buea, Box 63, Buea, Cameroon; Faculty of Medicine and Pharmaceutical Science, University of Douala, Douala, Cameroon; District Hospital Mamfe, South West Region, Mamfe, Cameroon; District Hospital Poli, North Region, Poli, Cameroon; Department of Obstetrics and Gynecology, Robert Debré Hospital, 75935 Cedex 19 Paris, France

**Keywords:** Teenage/adolescent pregnancy, Prevalence, Perinatal outcomes, Low birth weight, Preterm, Perineal tear

## Abstract

**Background:**

Teenage pregnancy is a high-risk condition that requires skilled antenatal care for good outcome. World estimates in 2008 report about 16 million births to adolescent mothers, most of them occurring in low and middle-income countries. In Cameroon, about 12 % of all births are to adolescent mothers. This study determines the prevalence of hospital teenage deliveries in the Buea Health District and compares the delivery outcomes and demographic characteristics between pregnant teenage mothers (14–19) and adult mothers (20–29 years). We also identify factors associated with adverse pregnancy outcomes.

**Methods:**

We undertook a retrospective study of case files of patients who gave birth in the Buea Regional Hospital during the period 2009–2012, to determine the prevalence of hospital-delivered teenage pregnancies in the BHR. We also undertook a, cross-sectional study to compare the outcomes of 148 singleton adolescent births with 360 adult births in three health facilities in the Buea Health District during the period March 1 to August 31, 2013.

**Results:**

The prevalence of teenage births was 13.3 %. The adverse fetal outcomes imputable to adolescent births were low birth weight (<2,500 g) (OR, 2.79; 95 % CI, 1.28-6.09), preterm babies (<37 weeks) (OR: 1.85; 95 % CI, 1.01-3.41), low 5 min Apgar score < 7 (OR: 1.66; 95 % CI, 0.91-3.0). Adverse maternal outcomes associated with teenage pregnancies were mainly perineal tear (OR, 1.6; 95 % CI, 0.95-2.7). Teenage births were not discovered in any significant way to cause preeclampsia/eclampsia, episiotomy, premature rupture of membranes and caesarean section. Maternal factors like age and gravidity were discovered to lead to adverse fetal outcomes in adolescents, while maternal factors like age, unemployment, marital status and gravidity were, for their part, directly responsible for adverse maternal outcomes in adolescents.

**Conclusion:**

Teenage pregnancies are quite prevalent in the Buea Health District, and hospital delivery common. Adolescent pregnancies are more likely to lead to adverse fetal and maternal outcomes than adult pregnancies.

## Background

According to the World Health Organization, teenage or adolescent pregnancy is a pregnancy occurring in girls aged 10 – 19 [1]. Teenage pregnancies constitute a serious health and social problem worldwide [2–4]. World estimates in 2008 report about 16 million births to adolescent mothers, most of them occurring in low and middle-income countries [5–7]. Among the social consequences of adolescent pregnancies are school drop-out, juvenile violence, suicide and sometimes homicide [6, 7].

Most studies establish a cause-and-effect relationship between teenage pregnancy and inadequate antenatal care [8–10], low birth weight, and preterm birth [5, 6, 11]. On the hand, such a relationship is less obvious in adverse pregnancy outcomes such as preeclampsia, cesarean delivery, vaginal instrumental delivery and postpartum hemorrhage [12].

Adolescent pregnancy constitutes a public health problem in Cameroon: 12 % and 9.3 % of all deliveries at the University Teaching Hospital (CHU) and Central Hospital Yaoundé respectively, are teenage [12–14]. Cameroon has one of the highest adolescent fertility rates in West and Central Africa. According to data collected in 2004 as part of the DHS, 22.7 % of adolescents under 20 were mothers of at least one child [15]. Cameroon’s adolescent fertility rate of 138 births per 1000 women aged < 19 is the highest in Central Africa. However, the country’s adolescent pregnancy rate is difficult to assess accurately, not least because national statistics on legal abortion are unreliable. They are so because of the wide differences in the local application of the law, and the discrepancies in public health data from one region of Cameroon to the other [15]. Statistics from the Social Welfare Centre in Buea, South West Region (SWR), Cameroon indicate that 5 % of girls in Buea terminate schooling every academic year because of pregnancy [16].

The aim of this study is threefold; namely, to 1) determine the prevalence of adolescent deliveries in the Buea Health District; 2) assess the effects of adolescent births on both mother and child; and 3) compare the effects of demographic characteristics (age, marital status, education) on delivery outcomes in teenage and adult pregnancies.

## Methods

### Study design

This was a cross-sectional study with retrospective and prospective phases.

### Study area

**Fig. 1:**
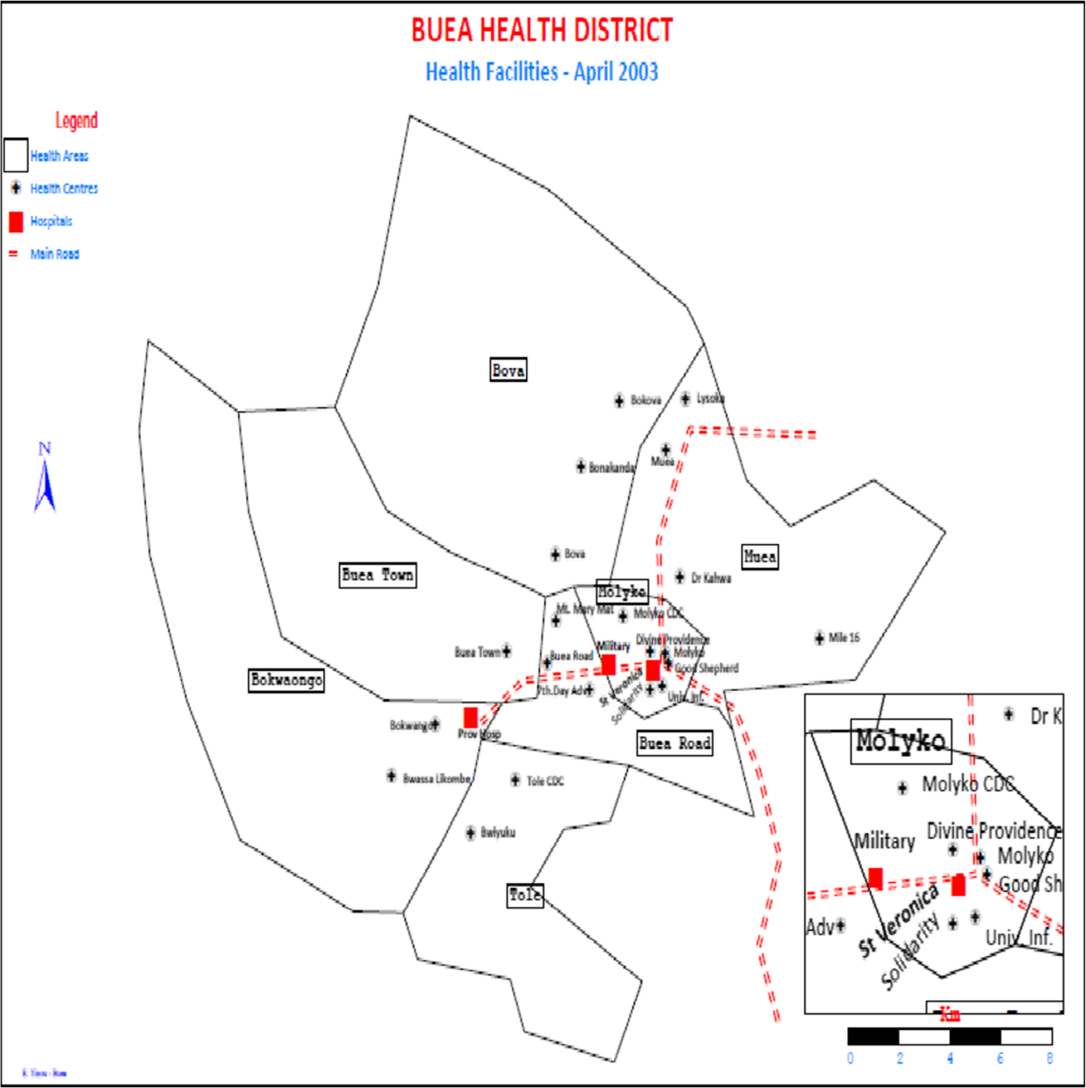
Buea Health District of the South West Region. Source: UPEC 2009

Buea is found in Fako Division in the South West Region of Cameroon. It covers a total surface area of 870 square km (Fig. 1). It has an equatorial climate, and temperatures range between 20-28 °C. The town experiences two major seasons; a rainy season that begins in March and ends in October, and a dry season that begins in November and ends in February. Annual rainfall varies from 3000 to 5000 mm.

### Study setting

The study was carried out in the Buea Regional Hospital (BRH), the Muea Health Centre, and the Buea Town Health Centre. BRH is a secondary care centre and the Muea Health Centre and the Buea town Health Centre both primary care centers. The Buea Health District was created in 1995 and has an estimated population of 76272. Midwives and state registered or assistant nurses carried out all deliveries in the centers under study while Obstetricians or General Practitioners did caesarean deliveries.

The study was conducted in two phases: a retrospective study carried out over a period of one month (February 1^st^ to 28^th^, 2013), and a prospective cross-sectional analytic case–control study carried out over a period of 6 months (March 1 to August 31, 2013).

All nulliparous teenagers aged 14–19 with a singleton pregnancy who delivered in the above-mentioned health facilities during the study period were enrolled as cases. The comparison group was taken among nulliparous women aged between 20 and 29, with a singleton pregnancy, and who delivered during the same period.

Excluded from the study were women with a history of any medical condition prior to the current pregnancy (e.g. hypertension, diabetes mellitus, or any cardiac, renal, endocrine or autoimmune diseases). Also excluded were women with no delivery records, women with pregnancies less than 28 weeks gestation. Adolescents < 14 were also excluded from the study given that they were particularly at risk because of immaturity of the pelvis. Nulliparous women above 30 and multifetal pregnancies were also excluded because they were already in the group of high-risk pregnancies (advanced maternal age in primigravida, and multifetal pregnancies in primigravida, respectively). Finally, women with any perinatal complication occurring after 48 hours were also excluded from the study.

### Sampling of participants

The Buea Health District comprises seven health areas that in turn have several maternity units. Of these seven health areas, five were selected randomly by balloting. For the retrospective study, each health area represented a stratum. Registers of the antenatal care department of each health facility were exploited. Count was kept of newly-registered teenagers who consulted and delivered during the study period. Adult women who delivered during the same period comprised the control group. After this phase, it was observed that, of the five health areas studied, three (Muea, Buea Town and Bokwango) handled most of the deliveries. The prospective cross-sectional analytic study was conducted in three health facilities within these health areas; namely, the Muea District Hospital, the Integrated Health Center Buea Town, and the Buea Regional Hospital. The participants in the selected health institutions were enrolled on admission for labor, interviewed with pre-tested questionnaires, and followed up till after delivery.

### Calculation of sample size

Sample size was calculated using the WHO-steps approach [17]. The minimum sample size was S = 384. A total of 508 participants (148 adolescents and 360 control) were enrolled for the study.

### Study procedure

After signing an informed consent form, participants were administered a pre-tested survey questionnaire. Socio-demographic information, mainly maternal age, gravidity, religion, marital status, number of antenatal visits, employment status and level of education, was obtained, as was information on maternal and fetal outcomes. Gestational age of pregnancy was derived from the last normal menstrual period. Occasionally we relied on the first trimester ultrasound scan in patients who came for ANC early in pregnancy.

The following maternal outcomes were used to compare between the cases and controls: preeclampsia-eclampsia, premature rupture of membranes, placenta previa, placenta abruption, perineal tear, and episiotomy. We did not record the degree of perineal tears because of inconsistencies in the classification across the different health facilities.

Fetal and neonatal outcomes compared between the two groups were prematurity, low birth weight, post term, 5 min Apgar scores < 7, stillbirth, and neonatal death. For the retrospective study, authorization to access medical records of patients was obtained from the directors of health facilities involved in the study.

### Ethical considerations

Ethical clearance was obtained from the Institutional Review Board (IRB) of the Faculty of Health Sciences (FHS), University of Buea; and Administrative authorization from the Regional Delegation of Public Health for the SWR.

### Data management and analysis

Data analysis was with Epi-Info version 7. Descriptive statistics was depicted using absolute numbers and simple percentages as appropriate. Regarding the cross sectional study, the frequencies for the entire variable were calculated and expressed as percentages of the total sample size. A bivariate analysis of categorical variables was done using Chi-square and Fisher’s exact tests (where appropriate) to compare between predictor variables and outcome variables. Univariable analysis was done by Mantel Haenszel method to compare predictor variables to the outcome. The results were reported as odd ratios (OR) together with their 95 % confidence intervals (CI). Multivariable analysis was done using logistic regressions, and only variables with a significance threshold of less than 0.1 were included in the final model. Results were reported as adjusted OR together with their 95 % CI. Measure of association was considered statistically significant at a two-tailed p-value when *P* < 0.05.

## Results

During the period 2010 – 2013 a total of 6564 women were registered for ANC at the BHD. Among this group, 13.32 % (874/6564) were teenagers.

Out of the 508 participants in the prospective study, 29.1 % (148/508) were pregnant. Most participants, 73.6 % (374/508) had a secondary level of education, and only 19.3 % had undergone tertiary education. Most of the participants, 96.9 % (492/508) were Christians. Among the study participants, 47.2 % (240/508) had employment, 58.7 % (298/508) were married, while 43.7 % (222/508) were nulliparous. Two hundred and ninety-six participants (58.3 %) attended 4 or more ANC visits throughout pregnancy. 52.4 % (266/508) participants had adverse perinatal pregnancy outcomes, while 43.2 % had adverse fetal outcomes. Of the 266 participants with adverse outcomes, 211 had adverse maternal outcomes.

### Comparison of socio-demographic characteristics

Table 1: It was observed that, the level of education was 3.7 times higher among adult mothers (*P* < 0.001) and adults were also 3.1 times more likely to be employed (*P* < 0.001). Furthermore, adults were 3.8 times more likely to be married and 3.0 times more likely to have more than one child (*P* < 0.001) compared to the teenage group.

**Table 1:** Comparison of socio-demographic variables among teenagers (14–19) and adult group (20–29) years

Variables	Teenagers (14–19 years) Cases (n = 148) N (%)	Control group (20–29 years) Cases (n = 360) N (%)	OR (95 % CI)	P-value
Education (years)				
Primary(6)	16(10.8 %)	20(5.6 %)	3.7(2.1-5.5)	<0.001
Secondary (7–13)	129(87.2 %)	245(68.1 %)		
Tertiary (≥14)	3(2.0 %)	95(26.4 %)		
Occupation				
Employed	43(10.8 %)	197(54.7 %)	3.1(2.0-4.7)	<0.001
Unemployed	105(70.9 %)	163(45.3 %)		
Marital status				
Married	53(35.8 %)	245(68.1 %)	3.8(2.6-5.7)	<0.001
Single	95(64.2 %)	115(51.9 %)		
Gravidity				
≥2	40(27.0 %)	246(68.3 %)	3.0(2.3-4.9)	<0.001
1	108(73.0 %)	114(31.7 %)		
Religion				
Christian	142(95.9 %)	350(97.2 %)	1.7(0.6-4.7)	<0.23
Muslim	6(4.1 %)	10(2.8 %)		
ANC				
≥4 Visits	60(40.5 %)	152(42.2 %)	1.02(0.7-4.6)	<0.4
0-3 Visits	88(59.5 %)	208(57.8 %)		

CI: Confidence interval

OR: Odds ratio

Religion and antenatal care (ANC) visits did not differ amongst the two groups (*P* < 0.2 and *P* < 0.4, respectively).

### Comparison of fetal outcomes

Table 2: Teenage mothers were more likely to have preterm babies (OR, 1.9; 95 % CI, 1.0-3.4: *P* < 0.03), LBW infants (OR, 2.8; 95 % CI, 1.3-6.1: *P* < 0.006) and low 5 min Apgar scores (OR, 1.7; 95 % CI, 0.9-3.0: *P* < 0.05) than adult mothers. On the other hand, the rates of neonatal deaths, stillbirths and post-terms were insignificant in both groups. Overall, there was more than a twofold increase in the likelihood of adverse fetal outcomes among teenage mothers (OR, 2.1; 95 % CI, 1.4-3.2: *P* < 0.001).

**Table 2:** Comparison of fetal outcome among teenagers (14–19 years) and adult group

Parameters	Teenagers (<20 years) (n = 148) N (%)	Control group (20–29 years) (n = 360) N (%)	OR (95 % CI)	P-value
Fetal Outcome				
Adverse	58(39.2 %)	84(23.3 %)	2.1(1.40-3.2)	<0.001
Normal	90(60.8 %)	276(76.7 %)		
Preterm				
Yes	20(13.5 %)	28(7.8 %)	1.9(1.0-3.4)	0.03
No	128(86.5 %)	332(92.2 %)		
Low birth weight(<2500 g)				
Yes	14(9.5 %)	13(3.6 %)	2.8(1.3-6.1)	0.006
No	134(90.5 %)	347(96.4 %)		
Post term				
Yes	4(2.7 %)	5(1.4 %)	2.0(0.5-7.5)	0.2
No	144(97.3 %)	355(98.6 %)		
5 min APGAR score < 7				
Yes	20(13.5 %)	31(8.6 %)	1.7(0.9-3.0)	0.05
No	128(86.5 %)	329(91.4 %)		
Still birth				
Yes	8(5.4 %)	10(2.8 %)	2.0(0.8-5.2)	0.08
No	140(94.6 %)	350(97.2 %)		
Neonatal death				
Yes	6(41.1 %)	9(2.5 %)	1.7(0.6-4.7)	0.2
No	142(95.9 %)	351(97.5 %)		
Malformation				
Yes	1(0.7 %)	2(0.6 %)	1.2(0.6-4.7)	0.2
No	128(86.5 %)	318(88.3 %)		
Placenta abruption				
Yes	4(2.7 %)	5(1.4 %)	2.0(0.5-7.5)	0.17
No	146(99.3 %)	358(99.4 %)		

CI: Confidence interval

OR: Odds ratio

### Comparison of maternal outcomes

Table 3: Adolescent mothers had a significantly higher rate of perineal tear compared with the adult population (OR, 1.59; 95 % CI, 0.95-2.67: P = 0.04). However, there were no significant differences between the two groups in the frequency of cesarean sections, eclampsia, pre-eclampsia, placenta previa, PROM and episiotomy. On the whole, there was a 1.5 times increase in the likelihood of adverse maternal outcomes among teenage mothers (OR, 1.5; 95 % CI, 1.3-2.4: P = 0.03)

**Table 3:** Comparison of maternal outcome among teenagers (14–19 years) and adult group

Parameters	Frequency (Percentage)	Teenagers (14–19years) (n = 148) N (%)	Control group (20–29 years) (n = 360) N (%)	OR (95 % CI)	P-value
Maternal Outcome					
Adverse		64(43.2 %)	123(34.2 %)	1.5(1.0-2.2)	0.03
Normal		84(56.8 %)	237(65.8 %)		
Pre-eclampsia					
Yes	10(4.7 %)	3(2.0 %)	7(1.9 %)	1.0(0.3-4.1)	0.5
No		145(98.0 %)	353(98.1 %)		
Eclampsia					
Yes	06(2.8 %)	3(2.0 %)	3(0.8 %)	2.5(0.5-12.3)	0.15
No		145(98.0 %)	357(99.2 %)		
PROM					
Yes	33(15.6 %)	11(7.4 %)	22(6.1 %)	1.2(0.6-2.6)	0.3
No		137(92.6 %)	338(93.9 %)		
Placenta previa					
Yes	05(2.4 %)	1(0.7 %)	4(1.1 %)	06(0.1-5.5)	0.4
No		147(99.3 %)	356(98.9 %)		
Perineal tear					
Yes	74(35.1 %)	28(18.9 %)	46(12.8 %)	1.6(1.0--2.7)	0.04
No		120(81.1 %)	314(87.2 %)		
Episiotomy					
Yes	12(5.7 %)	5(3.4 %)	7(1.9 %)	1.8(0.6-5.7)	0.2
No		143(97.3 %)	353(98.1 %)		
Cesarean section					
Yes	62(29.4 %)	20(13.5 %)	42(11.7 %)	1.2(0.7-2.1)	0.3

CI: Confidence interval

OR: Odds ratio

Preterm: Pregnancies < 37 weeks

Postterm: Pregnancies > 42 weeks

Adverse fetal outcomes: neonatal death, stillbirths, low 5 min Apgar scores, Preterm births and low birth weight infants

### Comparison of demographic parameters with adverse fetal outcomes

Table 4: Age (aOR, 2.79; 95 % CI, 0.05-0.83: *P* < 006) and gravidity (aOR, 9.08; 95 % CI 3.21-25.69) were significant factors in adverse fetal outcomes. Adverse fetal outcomes reduced with increasing maternal age and gravidity.

**Table 4:** Significant demographic parameters associated with adverse outcome

Parameter	Adjusted odd ratio (95 % CI)	P-value
Age (years)	2.79(0.05-0.83)	<0.006
Gravidity	9.08(3.21-25.69)	<0.001

CI: Confidence interval

### Comparison of demographic parameters with adverse maternal outcomes

Table 5: Age (aOR, 1.59; 95 % CI, 0.95-2.67: P = 0.04), employment (aOR, 0.48; 95 % CI, 0.29-0.82: P = 0.003), marital status (aOR, 0.58; 95 % CI, 0.36-0.96: P = 0.02) and gravidity (aOR, 9.08; 95 % CI, 3.21-25.69: *P* < 0.001) were significant factors in adverse maternal outcomes.

**Table 5:** Significant demographic parameters associated with adverse maternal outcome

Parameter	Adjusted odd ratio (95 % CI)	P-value
Age (years)	1.59(0.95-2.67)	0.041
Employment	0.48(0.29-0.82)	0.003
Marital status	0.58(0.36-0.96)	0.017
Gravidity	9.08(3.21-25.69)	<0.001

CI: Confidence Interval

## Discussion

Our study, set in the Buea Health District (BHD), a sub-urban region of Cameroon, sought to 1) determine the prevalence of adolescent hospital deliveries in the Buea Health District; 2) assess the effects of such births on both mother and child; and 3) compare demographic characteristics (age, marital status, education) between teenage mothers and comparison group of adults between 20–29.

The prevalence of teenage births in this study was 13.3 %. There were more adverse fetal and maternal outcomes among adolescent mothers between 14–19 than among adult mothers between 20–29. On the other hand, there was a close similarity between the demographic parameters and adverse outcomes in the two groups.

### Prevalence of teenage births

The 13.3 % prevalence of teenage pregnancies found among the study population fell within the 6-14 % range reported in other studies in Sub-Saharan African countries [9, 18–21]. It was higher than those of a similar study in Yaoundé [20, 22] though lower than the rate observed in the study conducted by Tebeu PM at al. [19], Sulaiman S at al. [22], Shah N et al. [23]. This high prevalence could be attributed to differences in socioeconomic status, level of education and level of employment.

Low levels of education, unemployment and pre-marital adventures were discovered to be important contributory factors to teenage pregnancy. These findings corroborated those reported by other studies [24–27].

### Socio-demographic characteristics

Reports concerning developing countries indicate an inverse relationship between education level and frequency of adolescent pregnancy and/or childbearing [28–31]. Regarding marital status, studies in low-income countries reported that most teenage pregnancies occurred within marriage and so did not occasion any social stigma [3]; but our study proved the contrary. We discovered instead that more teenage pregnancies occurred outside marriage than in it. This finding is confirmed by Kongnyuy J et al. and Ekachai K et al. [8, 32, 33]. Whatever contradiction there is between our study and those carried out in other low-income countries could be explained by the small sample size in our case and the socio-cultural differences from one situation to the other. Our study also shows that 73 % of adolescent pregnancies were in their first gestation while in the control group the percentage was 31.7.

A non-statistical significant trend was observed among 148 teenage mothers who gave birth during the study period. 59.5 % (88/146) of them had inappropriate antenatal care (less than 4 ANC). This trend was similar to that reported in other studies [32, 33]. Generally, it has been reported that about 72.8 % adolescents compared with 89.29 % women aged 20–29 attended four or more ANCs during pregnancy in Cameroon [32]. In the Philippines, among pregnant girls < 18, only 29 % received antenatal care, compared with 81 % among women aged 20–30 [11, 18]. This could be explained by the fact that most of the adolescents were unmarried, unemployed and less educated. Complications among adolescents were attributed to insufficient or inadequate antenatal care. Most of the health problems associated with adolescent pregnancies and childbearing could be prevented and controlled with timely and appropriate care during and after the pregnancy [34, 35].

### Maternal and fetal outcomes of teenage pregnancies

This study found more adverse maternal and fetal outcomes among adolescent mothers (14–19) than among adult women (20–29), a situation very much similar to those reported in literature on the subject [33, 36, 37]. However, our findings differ from those of McAnarney ER et al., [38], Elster A [39]. This difference can be explained by the fact that the various adolescent populations tend to be tributary to different socio-economic conditions and institutional constraints. Besides, the sample sizes are often very uneven.

Many studies have reported that adolescents are exposed to such adverse fetal outcomes as fetal death, neonatal death, low birth weight, IUGR and prematurity [40–42]. This study shows that pregnant adolescents had higher rates of LBW infants, premature babies and lower 5 min Apgar scores (<7) than adult mothers. This tied in with other studies [19, 24, 41]. However, the rates of post-term, neonatal deaths and stillbirths were similar between the two groups [23]. This may be explained by the fact that our study was not designed to detect rare outcomes and risk factors such as smoking or alcohol abuse in our adolescent population.

There was a significantly higher rate of perineal tears (18.9 %) among adolescent mothers in this study. This finding agrees with previous studies [14, 43, 44] but differs with them when it comes to preeclampsia/eclampsia, episiotomy, cesarean deliveries and PROM. Studies in Turkey confirm our position [45]. The widely-held belief that the biological immaturity of the adolescent pelvis causes cephalo-pelvic disproportion (CPD) leading to increased cesarean sections [46] was not confirmed by this study, probably because of the small sample size studied. Similarly, Imir did not find higher cesarean sections among adolescents than in the adult control group [45]. The controversy over this finding may be explained by the fact that adolescents generally give birth to small-size babies and so CPD is not necessarily a major problem in this age group. But Ogelle OM et al. found an increase in cesarean sections among adolescents in Nigeria [47]. It was also noted that although cesarean delivery was not statistically significant among adolescents, a greater proportion of them, 13.5 % underwent cesarean delivery than women in the comparison group (11.7 %) and the main indication was CPD.

It has been reported that teenage mothers were more likely to die from pregnancy and delivery complications [48–51]. However, we did not record any maternal deaths among teenage mothers in this study.

Our study noted that maternal age and gravidity had significant bearings on fetal outcomes. Furthermore, there was a nine-fold possibility of having adverse fetal outcomes among teenage mothers. Similar findings were reported by Kongnyuy et al. [32]. This may be explained by the fact that the small birth canal, rigid perineum and inappropriate psychological preparation for labor and delivery usually encountered among teenage primigravidas could lead to prolonged labor, with consequent fetal distress and low Apgar scores. Marital status had little or no effect on fetal outcomes, especially adverse, in our study.

Maternal age, marital status, unemployment and gravidity were seen to impact significantly on maternal outcomes. Gravidity was about nine times more of a contributory factor in maternal outcomes. The rigid perineum of primiparous mothers could be responsible for the perineal tears. The frequently inadequate prenatal care in adolescents has been reported previously [17, 32, 33] and may be explained by poverty, inadequate support from families and community, and even the judgmental attitude in prenatal care. Another study in Nigeria showed that unemployment impacted maternal outcomes [20].

There is very little health insurance coverage in Cameroon. Only a thin minority of company workers enjoy it. Therefore, most teenage and even adult pregnant women shoulder the cost of their antenatal care and delivery (including transportation, consultation, laboratory tests, and medication). This has usually been a burden to these patients who generally have low purchasing power or, as is often the case, no purchasing power at all.

### Study limitations

This was a hospital-based study with a small sample size; as such, we were not able to study the pregnancy outcomes of teenage mothers in the entire BHR. Socio-economic, sociocultural and psychological factors that could be an important determinant in pregnancy outcomes were not investigated in this study. There was no significant scope for follow-up, so outcomes like neonatal infections could not be investigated. We also did not study the effect of sex education among the teenage group.

## Conclusion

There was a high prevalence of teenage pregnancy in the BHD. Adolescent pregnancies were directly responsible for both maternal and fetal adverse outcomes.

The adverse fetal outcomes attributed to adolescent pregnancies were low birth rate (<2,500 g), low 5 min Apgar score (<7) and preterm births (<37 weeks). The rate of stillbirths and post-term deliveries was not significantly different between adolescent and adult mothers.

The adverse maternal outcomes caused by adolescent pregnancies were mainly perineal tear. Incidence of preeclampsia/eclampsia, episiotomy, placenta previa, cesarean section and PROM was not significantly different between adolescents and adult women.

Teenage mothers were more likely to be unemployed, unmarried and less educated than the control group. Demographic factors such as age, gravidity, marital status, and occupational status were contributory factors to adverse pregnancy outcomes.

### Recommendation

Similar studies should be carried out in other parts of Cameroon to obtain national statistics that could reveal the magnitude of the problem and so help policy makers in their search for curative strategies.
